# Congenital tibial pseudarthrosis: A challenge in pediatric radiology

**DOI:** 10.1016/j.radcr.2024.03.045

**Published:** 2024-04-04

**Authors:** Valentina Cariello, Maria C. Smaldone, Adele Durante, Paolo Pizzicato, Antonio Rossi, Rocco Minelli, Dolores Ferrara, Francesco Esposito, Massimo Zeccolini, Eugenio Rossi

**Affiliations:** a Department of Precision Medicine, University of Campania “Luigi Vanvitelli”, Piazza Miraglia, Naples 80138, Italy; b Department of Radiology, AORN “Santobono-Pausilipon”, Via Posillipo 226, Naples 80123, Italy; c University “Campus Biomedico”, Via Álvaro del Portillo 21, Rome 00128, Italy; d Department of Radiology, University of Molise “Unimol”, Via Francesco De Sanctis 1, Campobasso 86100, Italy

**Keywords:** Congenital tibial pseudarthrosis, Pediatric radiology, Skeletal system, X-rays

## Abstract

Congenital pseudarthrosis of the tibia (CPT) is a rare disorder affecting the skeletal system in pediatric population with an estimated incidence of 1:140,000 to 1:250,000 newborns. It is characterized by deformity of the tibia, including anterolateral bowing of the bone diaphysis and/or narrowing of the medullary canal, leading to instability or fracture. CPT can be either idiopathic or associated with underlying conditions such as type 1 neurofibromatosis (NF1), fibrous dysplasia, or Campanacci's osteofibrous dysplasia. Diagnosis is based on clinical and imaging findings, using conventional radiography and magnetic resonance imaging (MRI). The disorder is characterized by recurrent pathological fractures of the tibia or fibula during childhood, often beginning by the age of 2 years. Treatment options include surgical and nonsurgical management.

## Introduction

Congenital pseudarthrosis of the tibia (CPT) is a rare bone disorder affecting the lower limbs in neonates and infants. It manifests with a spectrum of deformities, encompassing outward bowing of the tibia (antero-lateral tibial angulation) and non-union of fractures (inability to heal).

The physiopathology remains unclear, although potential contributors include genetic predisposition (e.g., neurofibromatosis type 1), fetal constraint, developmental nutritional deficiencies, and early embryonic neurovascular anomalies [1]. In affected individuals, genetic defects and/or periosteal abnormalities, such as fibrous hamartoma, may hinder bone formation due to an imbalance between excessive osteoclastic activity and insufficient osteogenesis [1–5].

Two main forms of CPT have been distinguished: primary or neonatal form, which is evident at birth or within the first few weeks of life, and a secondary form manifesting later during childhood, as weight-bearing begins with ambulation [1]. Furthermore, CPT can be categorized based on imaging findings, guiding treatment decisions. Radiography represents the main imaging modality for CPT diagnosis, to assess the severity of bowing, bone loss, and the presence of fractures. Radiographic features enable the differentiation between cystic and dysplastic forms of the condition [1–3,6,7]. Therapeutic intervention for CPT presents significant challenges. The ongoing growth of the pediatric skeleton impedes stable fixation, while the inherent malformations of the tibial segments further compromise healing potential. Additionally, postsurgical stress on the repaired bone, even after skeletal maturity, can lead to refracture [1]. Consequently, a universally accepted approach is not feasible, necessitating individualized treatment plans based on disease severity: nonsurgical approaches like bracing can be used to prevent fractures in milder cases, while surgical intervention is often necessary for fractures or significant deformities [5]. Despite successful treatment, the underlying skeletal abnormalities predispose patients to a high risk of refracture. Repeated surgeries and long-term complications, including leg length discrepancies, knee malalignment, and even hip issues, may arise over time [3,5,12].

## Case Presentation

A newborn, 10-days-old and delivered via elective C-section (APGAR 8/9), has been transferred to our hospital's neonatal intensive care unit (NICU) due to severe respiratory distress that manifested a few hours after birth.

The pregnancy was uneventful, marked by normal fetal ultrasounds and negative results on the noninvasive prenatal testing (NIPT) for chromosomal aneuploidies and major microdeletions. Blood tests indicate a slight increase in C-reactive protein (CRP), while both chest X-ray and abdominal ultrasound show no abnormalities.

Echocardiography reveals a patent foramen ovale (PFO) with a mild shunt.

A diagnosis of resolved acute respiratory distress syndrome (ARDS) is established after approximately 48 hours of supportive therapy, including mechanical ventilation and antibiotic treatment.

**Fig. 1:**
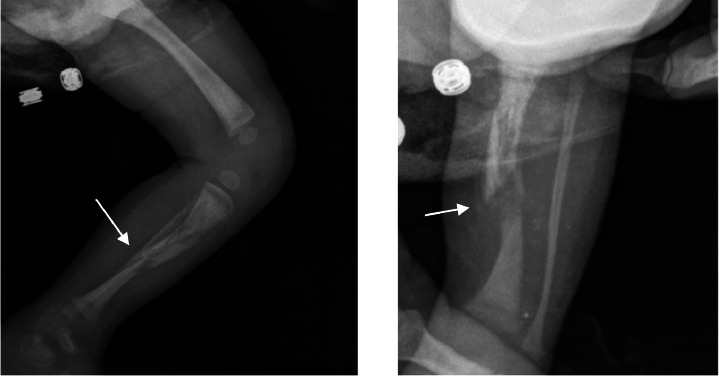
X-ray of the left lower limb identifies a mid-diaphyseal tibial fracture (white arrow).

Upon transfer to the nursery, the newborn exhibits inconsolable crying and reduced mobility in the left lower limb. An X-ray identifies a mid-diaphyseal tibial fracture (Fig. 1).

The mother denies any history of trauma.

The limb is promptly casted, and the patient is returned to the NICU.

**Fig. 2:**
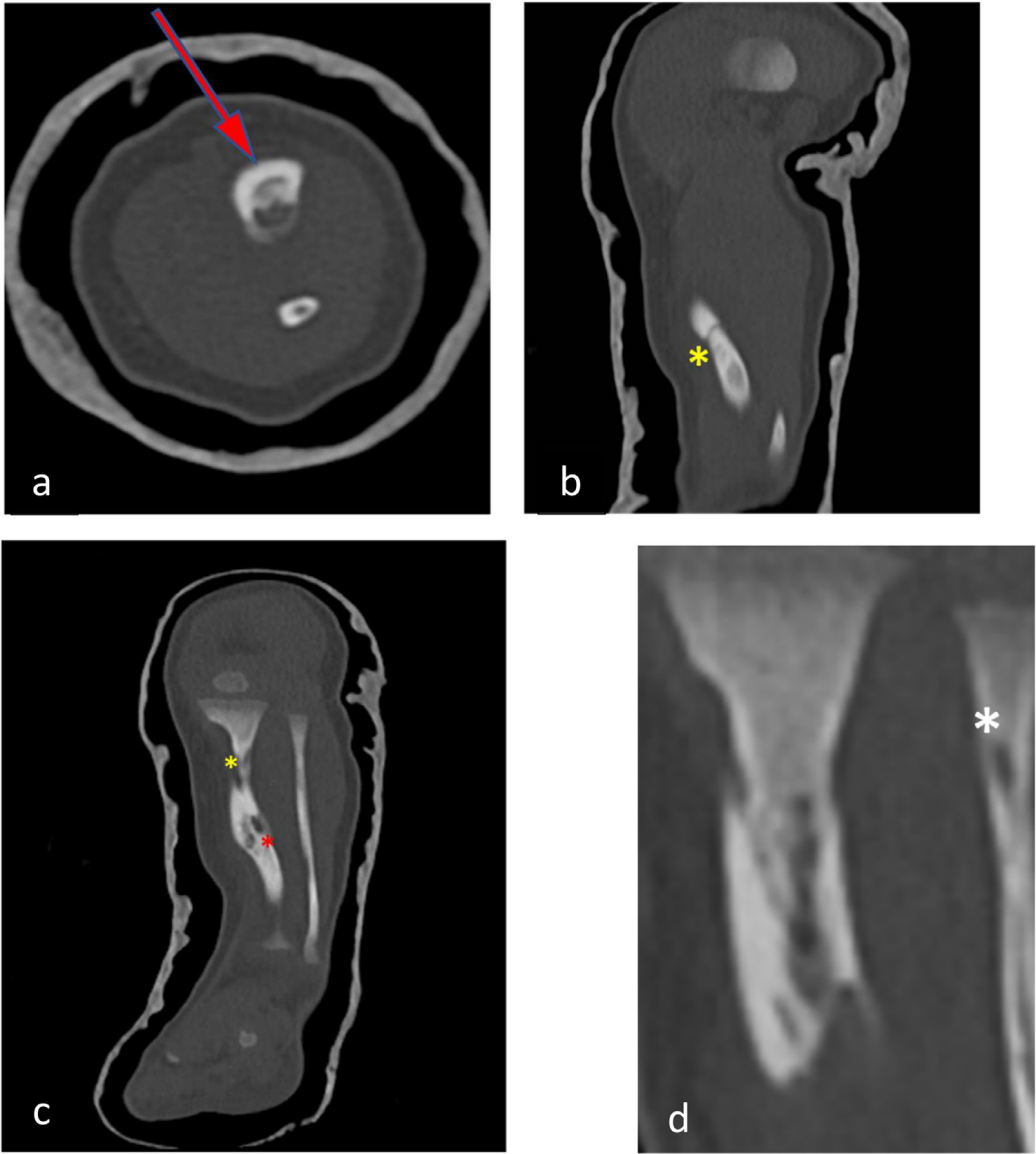
CT scan of the left lower limb shows (A) thickening (arrow) and (B and C) discontinuities of the bone cortex in the mid-diaphyseal regions of the tibia (yellow asterisks). (C) Multiple irregular hypodense areas are present along the tibia, extending to the bone cortex (red asterisk). (D) The fibula exhibits a small area of hypodensity in the proximal diaphyseal region, altering the cortical profile (white asterisk).

**Fig. 3:**
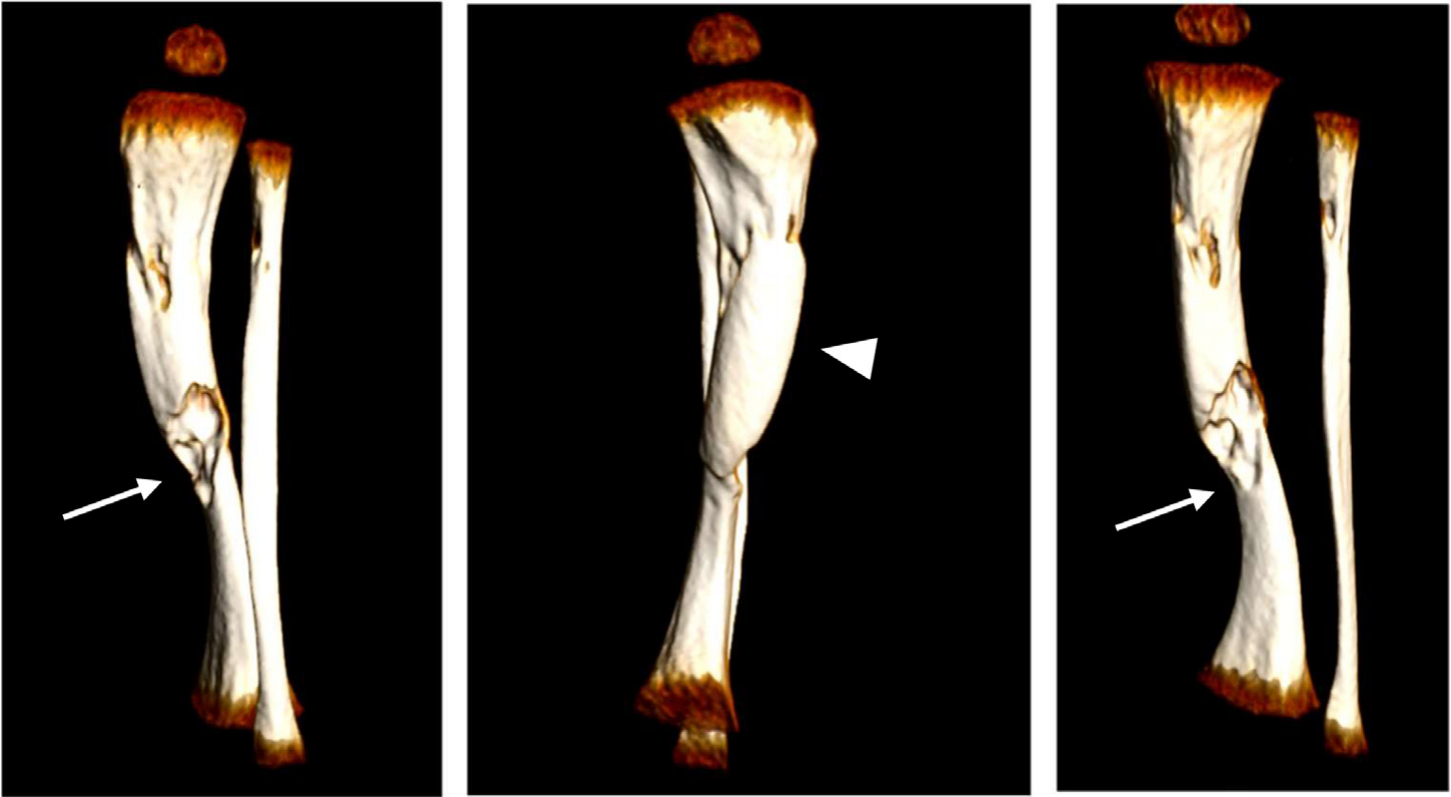
3D reconstructions also depict the morphologic and structural changes in tibia and fibula, demosrating the presence of osteolithic areas in both bones (arrows), along with tibial cortex thickening (arrowhead) .

A decision is made to conduct a CT scan of the lower limb, revealing a morphostructural alteration of the left tibia, with curvature in the distal diaphyseal segment (Figs. 2 and 3).

In the mid-diaphyseal regions of the tibia, there are evident discontinuities in the bone cortex, which also appears thickened. Simultaneously, multiple irregularly distributed hypodense areas are present along the tibia, extending to the bone cortex. The fibula, also curved, exhibits a small area of hypodensity in the proximal diaphyseal region, altering the cortical profile. No CT scan alterations are observed in the surrounding soft tissues.

Considering the imaging findings along with clinical and anamnestic data, there is a suggestion of an osteodysplastic condition of the leg, specifically leaning towards congenital tibial pseudarthrosis.

The imaging findings suggest the necessity for genetic counseling to rule out genetic disorders, particularly any association with NF1. The genetic counseling session indicates the absence of phenotypic anomalies and no visibly apparent café-au-lait spots.

The NGS analysis of genes linked to rasopathies, fibrous dysplasia, and osteogenesis imperfecta produces a negative result.

In accordance with a unanimous specialist consensus, neither an MRI nor a biopsy is deemed necessary. A leg brace is applied, and surgery is pending.

**Fig. 4:**
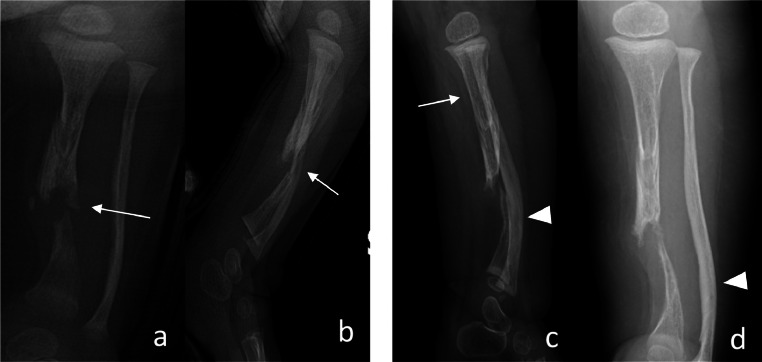
X-rays performed after 2 months (A and B) and after 8 months (C and D) show absence of osseous consolidation of the fracture (white arrows). (C and D) Along with fracture, tibial erosion is visible in its proximal tract (white arrow); fibula appears intact, but with significant antero-lateral bowing (arrowhead).

**Fig. 5:**
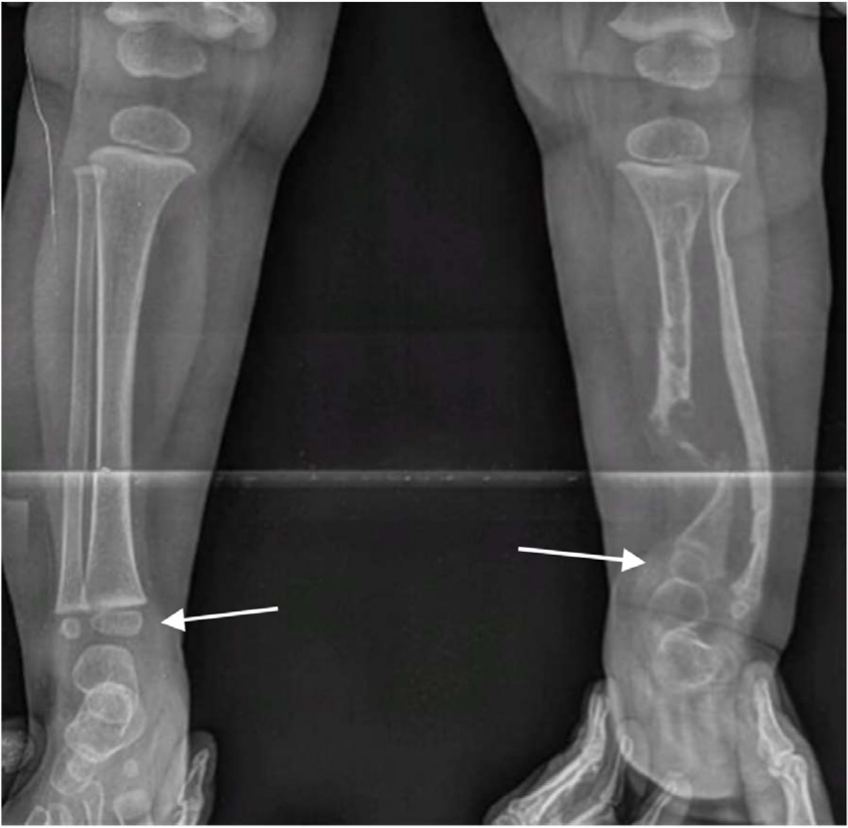
Lower limbs X-rays, performed during follow-up, show left leg 1.5 cm shorter than right (arrows).

During the follow-up, our diagnostic hypothesis gains further support as there is the absence of osseous consolidation of a fracture, and characteristic features of CPT become evident (Figs. 4 and 5).

**Fig. 6:**
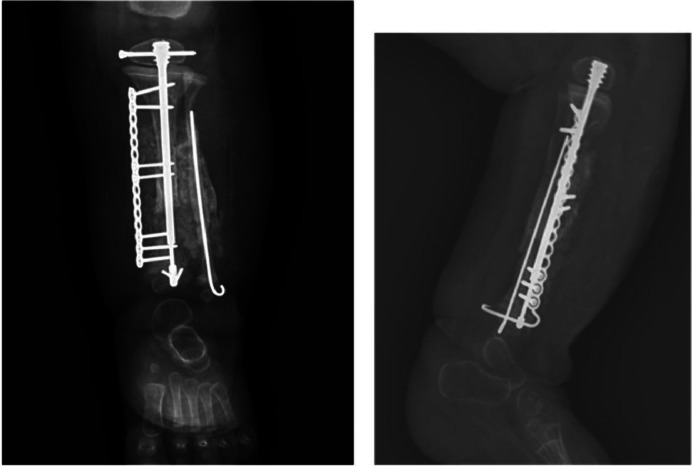
Radiograph of lower limb obtained after cross-union surgery shows good healing progress with properly positioned fixation implants.

Therefore, the patient at age of 12 months undergoes cross-union surgery, with evidence of good healing progress (Fig. 6).

## Discussion

Congenital pseudarthrosis of the tibia (CPT) is a rare and challenging disorder of the skeletal system, affecting pediatric population with an estimated incidence of 1:140,000 - 1:250,000 newborns [2,3,4]. The clinical onset of the disease is either at the time of birth or within the first 2 years of life, although it rarely remains undetected until puberty [3,8].

Even though its physiopathology has not been conclusively clarified yet, CPT can be defined as a bone metabolism disorder of the middle-distal part of the diaphysis, due to an altered regenerative and homeostatic capacity of the periosteum [5]. Decreased responses to bone morphogenic protein-2 (BMP2) are probably responsible for increased osteoclastic activity and decreased osteoblastic proliferation in the periosteum [2–4,5]. Abnormal periosteal thickening (“tumor-like” appearance) is a common finding due to myofibroblast hyperplasia, which appears histologically as marked cuff of fibrous tissue, also called fibrous hamartoma, around pseudarthrosis [2,5]. This causes severe impairment of bone growth, progressive to atrophy, due to narrowing and occlusion of the subperiosteal vessels [4].

CPT is therefore characterized by deformity of the tibia, including anterolateral bowing of bone diaphysis and/or narrowing of the medullary canal around the deformed area, which culminates in instability or fracture [2,3,8,9] that either develops spontaneously or after a history of trivial trauma [8]. Osseous dysplasia may result in either complete nonunion of the tibial fracture (pseudarthrosis) or a simple anterolateral angulation of the bone [2,3,8].

Shortening of the limb may occur because of tibial bowing and decreased growth in the distal tibial epiphysis. The fibula is affected in one-third of the patients [8].

CPT can be either idiopathic or associated with an underlying condition such as type 1 neurofibromatosis (NF1), fibrous dysplasia or Campanacci's osteofibrous dysplasia [4,5,10].

Diagnosis is based on clinical and imaging findings.

Lower limb deformities, including varus positioning and curvation of the leg, a discontinuity between tibial segments, and limb length discrepancy, may be discovered during clinical examination in a newborn or an infant. CPT must be differentiated from benign posterolateral bowing, which is common in the pediatric population. In early childhood, when walking begins, instability may raise suspicion for pseudarthrosis [2,5,11].

When there is suspicion of an association with NF1, a thorough neurological and dermatological examination is mandatory to prevent the inadvertent omission of stigmata (café-au-lait spots, axillary freckling, and neurological abnormalities), because a significant proportion of individuals diagnosed with CPT are affected by NF1, with estimates ranging from 40% to 80% [2].

Upon confirming this diagnosis, genetic investigations should be carried out alongside the follow-up of potential additional issues (e.g., scoliosis development, intracranial, or intraspinal neurofibromas).

Conventional X-rays serve as the initial diagnostic tool. CPT mainly affects the middle and distal thirds of the tibial segment, without any preference for sex or side. The fibula is also affected in more than half the cases. Radiographic findings range from mild to severe abnormalities, including anterolateral bowing and true discontinuity, as well as evidence of bone resorption at the fracture sites [3,6,12].

Radiographic imaging may also help distinguish cystic and dystrophic forms [3], according to Andersen classification. In cystic forms, which are visible between 6 weeks and 1 year of age, the bone cortex is uninterrupted, condensed, and thickened in the concave part of the curvature, while the medullary canal is narrow, and a cystic image can be observed at the apex of the curvature. The deformity progressively worsens and eventually results in a transverse fracture when the cortex breaks. In dysplastic forms, bowing is apparent at birth, and pseudarthrosis may develop. Other classifications include the Crawford classification, which describes 4 types of CPT having in common an anterolateral bowing of the affected tibia, and the Boyd classification describing 6 different types of CPT. Deformity can resemble an "hourglass" due to the narrowing of the apex of the curvature [2,3,6,7].

Pseudarthrosis is characterized by recurrent pathological fractures of the tibia or fibula during childhood, typically beginning around the age of 2 [2,13]. The affected bones may exhibit either thin, atrophic, or wide, hypertrophic ends. Pseudarthrosis can sometimes be mistaken for a fracture, which can result in multiple surgeries and disheartening results.

MRI can be helpful to better depict the morphology of pseudarthrosis and adjacent soft tissues abnormalities, including periosteal thickening, soft tissues edema, and possibly neurofibromas in children affected by NF [2].

MRI assists in preoperative planning by allowing for precise delineation of excision borders and accurate assessment of subtle soft-tissue changes. Moreover, the use of intravenous contrast medium enables bone perfusion imaging, which can be helpful in evaluating vascularization defects in the bone and determining the extent of resection [1].

MRI also provides prognostic information such as the type of pseudarthrosis, bone union or nonunion, and the length of the pseudarthrosis [8].

The management of congenital pseudarthrosis of the tibia involves diverse treatment modalities, including bone-grafting, the Ilizarow method, electric stimulation, or amputation, contingent upon the specific disease type. Despite advancements in techniques such as vascularized fibular transfers and the Ilizarov method, achieving union in pseudarthrosis may necessitate multiple surgical interventions [2,6,8].

The risk of amputation, although reduced, is never eliminated. In instances without tibial fracture, standard care involves bracing to prevent fractures [4]. However, consensus is lacking regarding the optimal surgical approach, age for intervention, and the most effective strategy to prevent refracture when a fracture is present. While the possibility of never achieving osseous union is accepted, maintaining osseous union itself can pose challenges, leading to repeated surgeries and secondary changes such as foot deformity, leg-length discrepancy, knee malalignment, and even hip dysplasia [1,4,6,10,11]. Repeated surgeries may result in interrupted childhood, prolonged disability, and, in some cases, amputation.

## Patient consent

Informed written consent was obtained from patient's parents for the publication of the case report.
